# Mosquito Fauna and Spatial Distribution in an Atlantic Forest Area in Rio de Janeiro State, Brazil, Reveal a High Risk of Transmission of Yellow Fever and Other Arboviruses

**DOI:** 10.3390/tropicalmed7120410

**Published:** 2022-11-30

**Authors:** Rafaella Moraes de Miranda, Anielly Ferreira-de-Brito, Júlia dos Santos Silva, Alexandre da Silva Xavier, Shayenne Olsson Freitas Silva, Jeronimo Alencar, Ricardo Lourenço-de-Oliveira

**Affiliations:** 1 Laboratório de Mosquitos Transmissores de Hematozoário, Instituto Oswaldo Cruz, Fundação Oswaldo Cruz, Rio de Janeiro 21040-900, Brazil; 2 Laboratório de Diptera, Instituto Oswaldo Cruz, Fundação Oswaldo Cruz, Rio de Janeiro 21040-900, Brazil

**Keywords:** Culicidae, diversity, richness, Atlantic Forest, primary vectors, yellow fever

## Abstract

In 2017–2019, Brazil recorded its most severe outbreak of yellow fever due to the spread of the virus (YFV) in the country’s southeast. Here, we investigated mosquito fauna and the spatial distribution of species in a primatology center in the Atlantic Forest bioregion in Rio de Janeiro state to evaluate the risk of YFV transmission in distinct environments. Fortnightly mosquito collections were performed from December 2018 to December 2019 at 12 sites along a disturbance gradient from a modified environment to 400 m inside the forest. We used ovitraps, BG-Sentinel, and protected human attraction (PHA). A total of 9349 mosquitoes of 21 species were collected. The collection method strongly influenced the captured fauna, with species such as *Anopheles cruzii*, *Psorophora ferox*, *Runchomyia cerqueirai*, *Wyeomyia incaudata*, *Wy. theobaldi*, *Sabethes chloropterus*, and *Sa. albiprivus* only collected via PHA. Collections with ovitraps resulted in low diversity and richness, with *Haemagogus leucocelaenus* and *Hg. janthinomys/capricornii* predominating. The diverse local fauna and the abundance and ubiquity of the latter species, which are the primary vectors of YFV, indicated that this area was highly vulnerable to arbovirus transmission, especially yellow fever, highlighting the need for improved surveillance and vaccination coverage in human and captive endangered non-human primates.

## 1. Introduction

Arboviruses (arthropod-borne viruses) are the agents of some of the emerging and re-emerging diseases with the greatest impact on human health. Yellow fever is a mosquito-borne disease caused by the yellow fever virus (YFV), which occurs in Africa and the Americas in two main transmission cycles: urban and sylvatic. These are distinguished by the ecological nature of the transmission, or, more specifically, the mosquito vector species: the anthropic *Aedes aegypti* in urban areas and several sylvatic Aedini and Sabethini species in forested environments [1,2]. In South America, YFV has been maintained only in the sylvatic transmission cycle involving non-human primates (NHPs) and arboreal mosquitoes of the genera *Haemagogus* and *Sabethes,* where humans can acquire the infection during epizootics [2,3,4,5].

In 2017–2019, Brazil recorded its most serious outbreak when YFV spread in the Atlantic Forest in the southeast region, where it had not circulated for almost 80 years [2]. During this outbreak, a high number of epizootics were reported, hundreds of monkeys were killed, and some NHP species were eradicated from some forest fragments and environmental conservation areas. Moreover, 2170 confirmed human infections with 932 deaths were recorded [5]. 

*Haemagogus* spp. play a key role in the transmission of sylvatic yellow fever in South America, mainly due to its primatophilic habit, which facilitates its contact with viremic NHPs [6,7,8]. Two species were identified as the primary YFV vectors during the 2017–2019 outbreak: *Haemagogus janthinomys/capricornii* and *Haemagogus leucocelaenus* [8]. Among the species of the genus *Sabethes*, *Sabethes chloropterus* (Humboldt, 1819) and *Sabethes albiprivus* (Theobald, 1903) are commonly found in enzootic YFV foci and were found to be naturally infected with the virus. However, *Sa. chloropterus* has greater local epidemiological prominence and was considered a secondary vector of YFV in the Atlantic Forest bioregion [8,9,10].

Brazil has the greatest diversity of species in the world, with more than 20% of the total species on the planet [11]. The Atlantic Forest is classified as one of the five areas with the highest rates of endemicity and diversity of fauna and flora worldwide [12]. 

The occurrence of species depends on factors such as the structural heterogeneity of the biome, with more diverse habitat resources allowing for more species to coexist while also minimizing the effect of competition and, consequently, increasing local biodiversity. Variations in the number of species between communities can be represented and quantified in several ways, with the most common being through diversity indices [13,14].

The spatial distribution of mosquito species is mainly related to the choice of oviposition site, which is strongly influenced by climatic and environmental factors. In addition, some Culicidae species’ eggs can withstand a period out of water without hatching, which is a natural ability of mosquitoes that lay their eggs out of water. This ability, which can be influenced by extrinsic factors, allows these species to withstand periods of drought [7].

The triggering, spread, and severity of an outbreak of a zoonotic arbovirus, such as YFV, are multifactorial. In the case of the 2017–2019 YFV outbreak in southeastern Brazil, the main drivers identified were poor vaccination coverage; increased areas with conditions favorable for NHPs; mosquito population growth in some parts of the Atlantic Forest combined with the loss of natural habitats in others; human contact with the forest; and the ecological plasticity of vectors, such as the ability of sylvatic mosquitoes to bite beyond the forest limits [2]. This last factor may have an important role, as a high proportion of those infected during the outbreak did not report having entered the forest, though they lived near or approached forest fragments for leisure, resting, and working (planting, weeding, harvesting). The zones with intermediate levels of forest cover and high exposure to the forest edge were identified as being more prone to the occurrence of human infections by YFV [15,16,17].

In this study, we identified and described mosquito fauna and spatial distribution of mosquito species in a fragment of the Atlantic Forest in Rio de Janeiro (RJ) state impacted by the 2017–2019 outbreak to evaluate the risk of YFV infections in distinct environments.

## 2. Material and Methods

The study protocol for the collection, capture, and transport of biological material was approved by the Brazilian Ministry of Environment’s Instituto Chico Mendes de Conservação da Biodiversidade (ICMBio-SISBIO 52472-2). All team members were vaccinated against YFV.

### 2.1. Study Area

**Figure 1:**
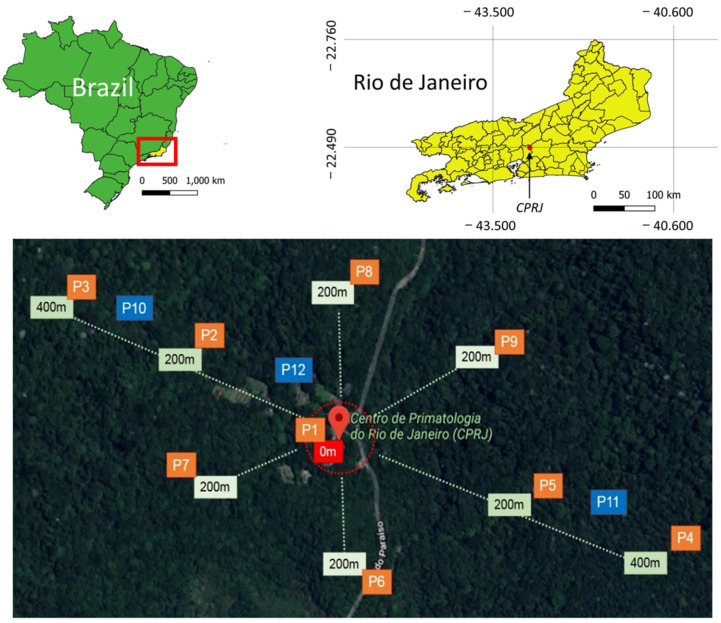
Arrangement of mosquito sampling sites in the Centro de Primatologia do Rio de Janeiro, Rio de Janeiro state, Brazil. The maps were prepared using QGIS 3.14.16 software and edited in Adobe Photoshop CS5. Reprinted from QGIS 3.14.16, a program under a CC BY license, with permission from Jeronimo Alencar—Fiocruz, original copyright 2022.

The survey was carried out at Centro de Primatologia do Rio de Janeiro (CPRJ) (22°29′46.5″ S, 42°54′31.7″ W), which is a breeding and research center for endangered New World NHPs located in the foothills of a coastal mountain chain called Serra do Mar in the municipality of Guapimirim, RJ, next to the former Estação Ecológica Estadual do Paraíso. The center is currently part of the Três Picos State Park and is located approximately 100 km from the city of Rio de Janeiro (Figure 1). This site was chosen because it is a modified environment surrounded by the Atlantic Forest and because of the continuing presence of NHPs. This latter consideration was due to the primatophilic behavior of the YFV mosquito vectors, as several of the NHP species affected by the 2017–2019 epizootics recorded in RJ were permanently kept in outdoor enclosures where they were exposed to mosquito bites and because the center was simultaneously the site of a study into the safety of YFV vaccination in endangered NHP species [18].

Mosquito captures were carried out at 12 sampling sites (P1–P12) located along transects that encompassed environments with different forest coverage, from an open field and the modified environment where primate enclosures and other facilities are located to inside the forest. One main transect was traced with five collection sites (P1–P5) established every 200 m. The collection site located in the middle (P1) served as a reference for two more transects, along which four additional sites were placed (P6–P9), each 200 m away from P1. Site P1 was located in the modified environment at the approximate geographic center of the CPRJ; P2 and P5–P9 were located within a radius of 200 m from P1 in an ecotone between the modified environment and the forest edge, while P3 and P4 were located inside the forest, 400 m from P1 (Figure 1).

In five sites (P1–P5), we used traps for the collection of adults (BG-Sentinel), as well as for immatures (ovitraps). Only ovitraps were used at four sites (P6–P9). In addition, the active capture of adults through protected human attraction (PHA) was conducted at three sites designated P10–P12. P10 and P11 were located inside the forest, while P12 was in the modified environment. 

From December 2018 to December 2019, ovitraps [19] containing two wooden paddles were hung from tree branches at a height of 2 m. The paddles and water were changed every 15 days. A total of 36 ovitraps were distributed equally from P1 to P9. The immature forms found in the water of the ovitraps were reared to adulthood in the laboratory. The paddles were examined under a stereomicroscope to search for mosquito eggs. The positive pallets were stored in humid chambers in an insectary in controlled temperature, humidity, and photoperiod conditions (26 ± 1 °C, 70 ± 10% RH, cycle of 12 h of light and 12 h in the dark) for 7–10 days. Then, the paddles were immersed in dechlorinated water to stimulate the eggs to hatch. Five alternating cycles of immersion and drying were done with drying intervals of four days. Species identification was performed after the emergence of adults. 

Mosquito capture via PHA was carried out with manual suction tubes, and all individuals used personal protective equipment to prevent mosquito bites. We used CO_2_ released by dry ice as an attractant for the adult collection with the BG-Sentinel trap. Captures of adult mosquitoes were carried out during four consecutive days per month, with collections taking place over four months in the rainy season (December 2018 to March 2019) and four months in the dry season (June to September 2019). During the four days of monthly collections, the BG-Sentinel traps were operated simultaneously in five collection sites (P1–P5) (Figure 1), and the captured mosquitoes were recovered every day at 6 PM when the dry ice and batteries were replaced. In the PHA captures, pairs of individuals collected mosquitoes for 40 min per sampling site, rotating between the points from 10 AM to 12 PM and again from 2 to 4 PM.

Adult mosquitoes captured with BG-Sentinels and PHA or raised in the laboratory from immature forms collected in ovitraps were immediately killed using dry ice and kept at −80 °C until tested for YFV.

The identification of mosquitoes was carried out from the direct observation of morphological characters under a stereomicroscope (Zeiss^®^, Aalen, Germany) on a cold table following Arnell (1973), Consoli and Lourenço-de-Oliveira (1994), Forattini (2002), Marcondes and Alencar (2010), and Abreu et al. (2019) [6,8,20,21,22].

### 2.2. Screening for YFV Natural Infections 

Adult mosquitoes were treated and tested for the presence of the YFV genome as previously described [8]. This analysis was almost exclusively carried out on non-blood-fed Aedini and Sabethini female mosquitoes caught with BG-Sentinel and PHA. Briefly, mosquitoes belonging to the same species and sampling site and date were pooled (≤10 individuals each), homogenized in 1000 µL of L-15 culture medium supplemented with 4% fetal bovine serum, and centrifuged at 10,000× *g* for 10 min at 4 °C. RNA was extracted from the supernatant by using the QIAamp Viral RNA Mini Kit according to the manufacturer’s instructions. The extracted RNAs were tested in duplicate via RT-qPCR using the set primers and protocols described before [8,23,24].

### 2.3. Statistical Analysis

Differences in mosquito community composition at each collection site were evaluated and compared using the Shannon–Wiener Diversity Index (H’) [25]. A t-test was performed with a significance level of 5% to assess the significant differences between the diversity indices using Past version 4.03 [26]. Ecological indices (abundance, richness, dominance, diversity, and Pielou equitability) were also calculated for each collection method (PHA, BG-Sentinel, ovitrap, pallet, and water) using Past version 4.03 [26]. The similarity between the sampling sites in terms of the number of species was estimated using the Sørensen qualitative similarity index (IS) based on the presence and absence of species. According to the general rule for similarity indices, values between 0.5 and 1.0 were considered high.

To assess the differences between mosquito populations at different capture sites and different capture methods, we used Kruskal–Wallis analysis with a significance level of 5% using IBM^®^ SPSS^®^ Statistics Version 23 (Armonk, NY, USA). Dunn’s post-test was used to assess which points and traps were statistically different from each other. Mann–Whitney analysis was used to evaluate the occurrence of significant differences between the different types of traps at the sampling points where only BG-Sentinels and ovitraps were placed, also using IBM^®^ SPSS^®^ Statistics Version 23.

## 3. Results

A total of 9349 mosquitoes were collected (Table 1). Just over half of this total (51.1%) was collected with ovitraps, with 5616 specimens reared from eggs (85.0%), and the remainder comprised the larvae and pupae found in the water (15.0%). Most of the 3733 adults (87.7%) were captured with the BG-Sentinel trap and the rest via PHA (12.3%).

Twenty-one species belonging to 12 genera were identified (Table 1). Most individuals of genera *Culex (Culex)* spp. and *Wyeomyia* could not be identified at the species level. Five species were collected using all the methods (ovitrap, BG-Sentinel, and PHA): the main YFV vectors *Hg. leucocelaenus* and *Hg. janthinomys/capricornii,* as well as *Aedes albopictus*, *Aedes terrens*, and *Limatus durhamii* (Table 1). On the other hand, some species were collected only with PHA captures: *Anopheles cruzii*, *Psorophora ferox*, *Runchomyia cerqueirai*, *Wyeomyia incaudata*, *Wyeomyia theobaldi*, and the secondary vectors of YFV *Sa. chloropterus* and *Sa. albiprivus*. *Ae. aegypti*, *Limatus pseudomethysticus*, and the predator *Toxorhynchites* sp. were only collected with ovitraps.

**Table 1:** Number and percentage of mosquito specimens collected across all sampling points and capturing methods at the Centro de Primatologia do Rio de Janeiro from December 2018 to December 2019.

Genus/Species	Adults						Ovitrap						Total	
	Protected Human Attraction		BG-Sentinel		Total		Paddle		Water		Total			
*Aedes aegypti* (Linnaeus)	0	0.0%	0	0.0%	0	0.0%	14	0.3%	0	0.0%	14	0.2%	14	0.1%
*Aedes albopictus* (Skuse)	8	1.7%	2	0.1%	10	0.3%	11	0.2%	2	0.2%	13	0.2%	23	0.2%
*Aedes scapularis* (Rondani)	59	12.9%	63	1.9%	122	3.3%	0	0.0%	0	0.0%	0	0.0%	122	1.3%
*Aedes terrens* (Walker)	4	0.9%	8	0.2%	12	0.3%	788	16.5%	0	0.0%	788	14.0%	800	8.6%
*Anopheles cruzii* (Dyar & Knab)	1	0.2%	0	0.0%	1	0.0%	0	0.0%	0	0.0%	0	0.0%	1	0.0%
*Culex urichii* (Coquillett)	0	0.0%	1	0.0%	1	0.0%	0	0.0%	444	52.7%	444	7.9%	445	4.8%
*Culex (Mcx.)* sp.	0	0.0%	0	0.0%	0	0.0%	0	0.0%	7	0.8%	7	0.1%	7	0.1%
*Culex* (*Culex*) spp.	41	9.0%	3030	92.5%	3071	82.3%	0	0.0%	0	0.0%	0	0.0%	3071	32.8%
*Haemagogus janthinomys* Dyar/*Hg. capricornii* Lutz	54	11.8%	3	0.1%	57	1.5%	509	10.7%	0	0.0%	509	9.1%	566	6.1%
*Haemagogus leucocelaenus* (Dyar & Shannon)	99	21.6%	17	0.5%	116	3.1%	3452	72.3%	13	1.5%	3465	61.7%	3581	38.3%
*Limatus durhamii* Theobald	11	2.4%	2	0.1%	13	0.3%	0	0.0%	60	7.1%	60	1.1%	73	0.8%
*Limatus pseudomethysticus* (Bonne-Wepster & Bonne)	0	0.0%	0	0.0%	0	0.0%	0	0.0%	311	36.9%	311	5.5%	311	3.3%
*Mansonia titillans* (Walker)	0	0.0%	6	0.2%	6	0.2%	0	0.0%	0	0.0%	0	0.0%	6	0.1%
*Mansonia* sp.	0	0.0%	1	0.0%	1	0.0%	0	0.0%	0	0.0%	0	0.0%	1	0.0%
*Psorophora ferox* (Humboldt)	1	0.2%	0	0.0%	1	0.0%	0	0.0%	0	0.0%	0	0.0%	1	0.0%
*Runchomyia cerqueirai* (Stone)	2	0.4%	0	0.0%	2	0.1%	0	0.0%	0	0.0%	0	0.0%	2	0.0%
*Runchomyia frontosa* (Theobald)	24	5.2%	27	0.8%	51	1.4%	0	0.0%	0	0.0%	0	0.0%	51	0.5%
*Runchomyia humboldti* (Lane & Cerqueira)	17	3.7%	18	0.5%	35	0.9%	0	0.0%	0	0.0%	0	0.0%	35	0.4%
*Runchomyia reversa* (Lane & Cerqueira)	17	3.7%	15	0.5%	32	0.9%	0	0.0%	0	0.0%	0	0.0%	32	0.3%
*Runchomyia* sp.	20	4.4%	19	0.6%	39	1.0%	0	0.0%	0	0.0%	0	0.0%	39	0.4%
*Sabethes albiprivus* Theobald	3	0.7%	0	0.0%	3	0.1%	0	0.0%	0	0.0%	0	0.0%	3	0.0%
*Sabethes chloropterus* (Humboldt)	21	4.6%	0	0.0%	21	0.6%	0	0.0%	0	0.0%	0	0.0%	21	0.2%
*Toxorhynchites* sp.	0	0.0%	0	0.0%	0	0.0%	0	0.0%	5	0.6%	5	0.1%	5	0.1%
*Tricoprosopon* sp.	0	0.0%	1	0.0%	1	0.0%	0	0.0%	0	0.0%	0	0.0%	1	0.0%
*Wyeomyia incaudata* (Root)	2	0.4%	0	0.0%	2	0.1%	0	0.0%	0	0.0%	0	0.0%	2	0.0%
*Wyeomyia theobaldi)* (Lane & Cerqueira)	26	5.7%	0	0.0%	26	0.7%	0	0.0%	0	0.0%	0	0.0%	26	0.3%
*Wyeomyia confusa* (Lutz)	0	0.0%	1	0.0%	1	0.0%	0	0.0%	0	0.0%	0	0.0%	1	0.0%
*Wyeomyia* spp.	48	10.5%	61	1.9%	109	2.9%	0	0.0%	0	0.0%	0	0.0%	109	1.2%
Total	458	100.0%	3275	100.0%	3733	100.0%	4774	100.0%	842	100.0%	5616	100.0%	9349	100.0%

The capture time differed between the three methods, with the ovitrap and the PHA operating for the longest and shortest time, respectively. Even so, the collection method with the greatest diversity and richness was PHA (H’ = 2.26 and 17 species), followed by BG-Sentinel (H’ = 2.02). The paddles of the ovitraps had the lowest diversity (H’ = 0.80) and richness (S = 5) and, consequently, a high level of dominance (D = 0.56), with the species *Hg. leucocelaenus* (n = 3452) and *Hg. janthinomys/capricornii* (n = 509) representing 72% and 11% of the total number of individuals collected, respectively (Table 2). However, the paddles had by far the highest abundance (n = 4774), or 78% of the total number of Culicidae collected and identified at the species level.

A total of 4774 adult mosquitoes were obtained from hatched eggs gathered from 2808 recovered paddles collected fortnightly over the 13 months of the study (26 collections), with 842 adults obtained from larvae and pupae found in the water of the ovitraps. The most abundant species among the immatures collected with ovitraps were *Hg. leucocelaenus* (61.7%), *Ae. terrens* (14.0%), and *Hg. janthinomys/capricornii* (9.1%) (Table 1). These species were the most abundant among the adults that emerged from the eggs found on the paddles (72.3%, 16.5%, and 10.7%, respectively). In contrast, of the larvae and pupae found in the water of the ovitraps, the most abundant species were *Culex urichi* (52.7%) and *Li. pseudomethysticus* (36.9%) (Table 1).

*Culex (Culex)* spp. were the most abundant (82.3%) across all collections of adults. However, the composition of the fauna and abundance changed when analyzing the results of adult collections according to the method used. *Culex (Culex)* spp. accounted for 92.5% of the total collected with BG-Sentinel with CO_2_ as an attractant, but only 9.0% of the mosquitoes captured with PHA, where other species were much more abundant, such as *Hg. leucocelaenus* (21.6%) and *Ae. scapularis* (12.9%) (Table 1).

The abundance of immature forms in ovitraps (Table 3) was significantly heterogeneous among the sampling sites (P1–P9; Kruskal–Wallis, *p* = 0.01), with P3 (inside the forest) having the highest abundance of specimens sampled with ovitraps, followed by P8 (situated roughly in the intermediate ecotone between the modified environment and the forest) (Table 3). The abundance of eggs collected with ovitrap paddles was greater than that of the larvae and pupae found in the water at all sampling sites except P6 (Table 3). Sampling sites P4 and P5 were similar in species composition and abundance, considering the specimens gathered with the ovitraps, but differed from the other sites. The Kruskal–Wallis analysis indicated significant differences in the abundances of mosquito specimens obtained with BG-Sentinel versus the ovitraps at P2, P4, and P5 (P2: *p* = 0.037, P4: *p* = 0.000, P5: *p* = 0.017). Dunn’s post-test indicated that the abundances in the BG-Sentinel at these three points differed from those of the ovitrap paddles and ovitrap water.

Adult mosquito collections made with BG-Sentinels had the greatest abundance at P5 (51.5%), located in the forest ecotone (Table 3), with a practically uniform distribution at the remaining sampling sites, ranging from 9.1% (P3) to 13.5% (P2), regardless of location, whether inside the forest or the modified environment. A similar pattern was found with captures made with PHA, with no significant difference (Kruskal–Wallis, *p* = 0.50) in the abundances between captures made inside the forest (P10 and P11) and the modified environment (P12). However, when analyzing the species composition by sampling site (Table 4), P5, which was located in the forest ecotone, had the highest number of mosquito specimens (n = 2047), while the greatest richness was recorded at two sites located inside the forest: P4 and P11. In contrast, the greatest diversities were registered in captures made with PHA at P11 (inside the forest) and P12 (in the modified environment). Furthermore, the highest evenness values were observed at the three collection points where captures with PHA were conducted (P10 to P12), regardless of the site environment. That is, there was a more similar distribution of the number of specimens of each mosquito species when captures were made with PHA, regardless of whether the sampling occurred in the open field or the forest (Table 4).

Although *Hg. leucocelaenus* was more abundant than *Hg. janthinomys*/*capricornii* across the study area (Mann–Whitney, *p* = 0.007), there was no significant difference in the abundances of both species between the sampling sites (Kruskal–Wallis, *p* = 0.204).

When we analyzed the similarity between the collection points (Sørensen’s similarity (IS)), we found that the highest similarity (IS > 0.81) was between the sites where captures were performed only with PHA (Table 5). This may indicate that the collection method influenced the composition of the captured fauna more than the sampling location. When comparing the points where there were collections of adults with BG and immature ones with ovitraps (P1 to P5), there was a high similarity for all paired comparisons (IS ranging from 0.59 to 0.83). Among these comparisons, it is interesting to highlight that particularly high similarity values were observed in comparisons between the modified environment (P1) and sites P3 and P4, which were located further into the forest (IS = 0.69 and 0.80, respectively).

A total of 287 adult mosquitoes caught with BG-Sentinel or PHA were screened for YFV, all of which were negative (Table 6). Almost 86% of tested specimens were *Hg. leucocelaenus* (n = 116), *Hg. janthinomys/capricornii* (57), and *Ae. scapularis* (73).

## 4. Discussion

Greater awareness of the diversity of mosquito species is fundamental for assessing possible changes in their behavior and adaptations according to the different environmental conditions in areas where the environment has been subjected to and/or is undergoing anthropogenic change [27].

In this study, 21 species of mosquitoes belonging to 12 genera were recorded in sites located on a disturbance gradient from the open and modified environment to within the Atlantic Forest at CPRJ. Intriguingly, this figure was considerably lower than the diversity of mosquitoes previously described in sites on the same slope of Serra do Mar. For instance, studies undertaken at Serra dos Órgãos National Park and Guapiaçu Reserve, located around 9 and 20 km from the CPRJ, recorded 44 and 59 mosquito species, respectively [28,29].

The collection method considerably influenced the results as they related to mosquito species diversity, richness, and abundance across the sampling sites. Species that do not breed in open containers, such as ovitraps, were not collected at points where only this collection method was used (P6–P9). The distribution of *Ae. scapularis* illustrated this finding since it was found only at sites where adult collections were performed, whether with BG-Sentinels or PHA. This behavior was also shared by the species of genera *Runchomyia* and *Wyeomyia*. The distribution of some species appeared to be affected more by the location of the sampling site, as was the case of the two *Sabethes* species, which were collected only at sites where captures were carried out with PHA (P10–P12). The use of ovitraps essentially selected species of tribe Aedini, such as *Hg. leucocelaenus*, *Ae. terrens*, and *Hg. janthinomys/capricornii* on the paddles, and *Culex urichii* and *Li. pseudomethysticus* in the water held in the ovitraps. Adults of *Culex* (*Culex*) spp. were abundant where BG-Sentinels were used (P1–P5) but accounted for only 9.0% of mosquitoes captured using PHA where, in contrast, primary and secondary YFV vectors, such as *Ae. scapularis*, *Hg. janthinomys/capricornii*, and *Hg. leucocelaenus*, were much more frequent. *Hg. leucocelaenus* represented 21.7% of mosquitoes captured with PHA, followed by *Ae. scapularis* (12.9%). The latter species was considered a secondary vector of yellow fever during the recent epidemic in Rio de Janeiro [8], was already found infected with YFV in other parts of Brazil [30,31], and was experimentally demonstrated to be able to transmit the virus between monkeys [32,33].

Interestingly, around 70% of immatures collected with ovitraps, regardless of their location, belonged to the two *Haemagogus* species considered to have been the primary YFV vector in RJ and other outbreaks in southeastern and southern Brazil [8,31,34,35,36,37,38,39,40].

Altogether, the results of adult and immature collections indicated that *Hg. leucocelaenus* is more abundant than *Hg. janthinomys*/*capricornii*. However, there was no difference in the abundance of both species across the sampling sites in the transects. That is, they occurred in similar abundance from the open field and modified environment to 400 m into the forest. This remarkable aspect of the species’ distribution had a major impact on YFV transmission.

These two vectors can move for several kilometers [41], covering forest and modified environments, a behavior that facilitates the spatial dissemination of the YFV and viral transmission from viremic NHPs living deep in the forest to humans, not only when they enter the forest or the forest edges but also in open fields and anthropic, modified environments with a certain proximity to the forest. Their ability to move from inside the forest, where they breed in tree holes and usually bite NHPs, to attacking humans in the intermediate ecotone or the modified environment was indicated as an important risk factor during the 2017–2019 YFV outbreak. Many people who became infected or died in this outbreak believed themselves unreachable by epizootic transmission because they lived in peri-urban areas and did not enter forests or their surroundings and consequently did not prioritize getting vaccinated [2]. However, the ability to fly a considerable distance [41] and the spatial distribution of *Hg. leucocelaenus* and *Hg. janthinomys*/*capricornii*, as observed in the CPRJ, illustrate how people can become exposed to YFV-infected sylvatic mosquito bites in rural, peri-urban, and even urban areas adjacent to the Atlantic Forest.

The failure to detect natural infections in the tested mosquitoes from the CPRJ area was consistent with the local epidemiological data. Our collections started in December 2018 and lasted until December 2019, a period during which no human cases were diagnosed in the entire transmission season from July 2018 to June 2019 across RJ. Moreover, the last suspected infections in NHPs were reported five months earlier (July 2018) and were considered remnants of the previous season. The only and last record of YFV circulation in the state was of a dead howler monkey found approximately 90 km from the CPRJ [24].

Our findings on mosquito diversity and spatial distribution at CPRJ, particularly concerning the primary and secondary vectors of YFV, also indicated that the captive NHPs were similarly exposed to their bites and the risk of mosquito-borne agents, including arboviruses, regardless of the location of the outdoor enclosures. The local fauna includes several species indicated as vectors of several arboviruses in Brazil [42]. Antibodies against some *Flaviviruses* and other arboviruses were reported in captive NHPs born at CPRJ [43]. Altogether, these data triggered the vaccination of NHPs against YFV with the YF 17DD attenuated virus, which was promptly initiated in 2018 at CPRJ to protect captive animals of susceptible endangered species and to aid in reducing transmission [18,44]. The protocol we used to test mosquitoes can detect both wild-type and vaccinal YFV [23]. The negative tests of wild mosquitoes collected in this study during the period of vaccination of the animals in the CPRJ corroborate the conclusions of Miranda et al. (2022) regarding a lack of evidence of uncontrolled transmission of this vaccine virus in nature from viremic New World NHPs [44].

## 5. Conclusions

Considering that the abundance of adult mosquitoes was practically uniform regardless of location and the greatest richness and diversity were recorded when captures were made via PHA, whether inside the forest or the modified environment, we concluded that the study area has a high potential for arbovirus transmission. This is particularly concerning due to the ubiquitous spatial distribution of primary and secondary YFV vector species, such as *Hg. leucocelaenus*, *Hg. janthinomys*, *Ae. scapularis*, and *Sa. chloropterus*, and even the occurrence of the urban vector *Ae. aegypti*. These findings highlight the need to improve monitoring for the emergence of febrile diseases and vaccine coverage in humans and captive NHPs of endangered species.

## Figures and Tables

**Table 2:** Ecological indices according to each mosquito collection method at the Centro de Primatologia do Rio de Janeiro from December 2018 to December 2019.

Ecological Indices	Protected Human Attraction	BG-Sentinel	Ovitrap	
			Paddle	Water
Richness (S)	17	13	5	5
Specimens	369	182	4774	830
Dominance (D)	0.14	0.18	0.56	0.43
Pielou equability (J)	0.80	0.79	0.50	0.60
Shannon’s diversity (H’)	2.26	2.02	0.80	0.97

**Table 3:** Absolute numbers and percentages of specimens collected at nine sampling sites at the Centro de Primatologia do Rio de Janeiro according to the type of collection from December 2018 to December 2019.

		Adults and Immatures					Immatures				Adults			Total
		P1	P2	P3	P4	P5	P6	P7	P8	P9	P10	P11	P12	
Ovitrap	Water	75	20	109	160	35	94	63	113	173	-	-	-	842
		8.9%	2.4%	12.9%	19.0%	4.2%	11.2%	7.5%	13.4%	20.5%	-	-	-	100.0%
	Paddles	534	573	1468	223	326	73	515	853	209	-	-	-	4774
		11.2%	12.0%	30.7%	4.7%	6.8%	1.5%	10.8%	17.9%	4.4%	-	-	-	100.0%
Adult captures	BG-sentinel	432	441	298	418	1686	-	-	-	-	-	-	-	3275
		13.2%	13.5%	9.1%	12.8%	51.5%	-	-	-	-	-	-	-	100.0%
	Protected human attraction	-	-	-	-	-	-	-	-	-	103	221	134	458
		-	-	-	-	-	-	-	-	-	22.5%	48.3%	29.3%	100.0%

**Table 4:** Distribution, richness, diversity, and equitability of mosquito specimens obtained in the immature and adult collections at the Centro de Primatologia do Rio de Janeiro from December 2018 to December 2019 at 12 sampling sites.

Species/Collection Point	P1	P2	P3	P4	P5	P6	P7	P8	P9	P10	P11	P12	Total
*Aedes aegypti*	0	0	12	0	0	0	0	2	0	0	0	0	14
*Aedes albopictus*	0	0	5	1	0	0	4	0	5	2	0	6	23
*Aedes scapularis*	10	19	6	4	24	0	0	0	0	15	19	25	122
*Aedes terrens*	361	6	301	26	14	0	84	0	4	0	3	1	800
*Anopheles cruzii*	0	0	0	0	0	0	0	0	0	0	1	0	1
*Culex urichi*	41	15	61	81	12	4	39	74	118	0	0	0	445
*Culex (Mcx.)* sp.	0	0	0	0	0	0	1	0	6	0	0	0	7
*Culex* (*Culex*) spp.	403	397	263	373	1594	0	0	0	0	5	23	13	3071
*Haemagogus janthinomys/capricornii*	29	74	78	50	82	0	83	95	21	13	22	19	566
*Haemagogus leucocelaenus*	147	498	1096	154	233	73	344	756	181	35	44	20	3581
*Limatus durhamii*	19	0	17	15	1	10	0	0	0	4	2	5	73
*Limatus pseudomethysticus*	15	4	18	66	21	80	23	37	47	0	0	0	311
*Mansonia titillans*	1	1	0	2	2	0	0	0	0	0	0	0	6
*Mansonia* sp.	0	1	0	0	0	0	0	0	0	0	0	0	1
*Psorophora ferox*	0	0	0	0	0	0	0	0	0	1	0	0	1
*Runchomyia cerqueirai*	0	0	0	0	0	0	0	0	0	1	1	0	2
*Runchomyia frontosa*	2	0	12	2	11	0	0	0	0	1	8	15	51
*Runchomyia humboldti*	0	2	5	0	11	0	0	0	0	7	4	6	35
*Runchomyia reversa*	2	2	0	1	10	0	0	0	0	0	10	7	32
*Runchomyia* sp.	4	6	0	7	2	0	0	0	0	7	7	6	39
*Sabethes albiprivus*	0	0	0	0	0	0	0	0	0	1	1	1	3
*Sabethes chloropterus*	0	0	0	0	0	0	0	0	0	3	15	3	21
*Toxorhynchites* sp.	0	1	0	0	2	0	0	2	0	0	0	0	5
*Tricoprosopon* sp.	0	0	0	1	0	0	0	0	0	0	0	0	1
*Wyeomyia (Pho.) incaudata*	0	0	0	0	0	0	0	0	0	0	0	2	2
*Wyeomyia (Pho.) teoboldti*	0	0	0	0	0	0	0	0	0	0	26	0	26
*Wyeomyia confusa*	0	0	0	1	0	0	0	0	0	0	0	0	1
*Wyeomyia* sp.	7	8	1	17	28	0	0	0	0	8	35	5	109
Total	1041	1034	1875	801	2047	167	578	966	382	103	221	134	9349
Diversity indices	P1	P2	P3	P4	P5	P6	P7	P8	P9	P10	P11	P12	
Richness (S)	13	14	13	17	15	4	7	6	7	15	17	16	
Specimens	1041	1034	1875	801	2047	167	578	966	382	103	221	134	
Shannon’s diversity (H’)	1.48	1.21	1.33	1.69	0.87	0.97	1.22	0.77	1.3	2.16	2.43	2.4	
Shannon’s equitability (J)	0.57	0.46	0.52	0.60	0.32	0.70	0.63	0.43	0.67	0.80	0.86	0.87	

**Table 5:** Sørensen similarity values (IS) between the mosquito sampling sites at the Centro de Primatologia do Rio de Janeiro.

IS	P1	P2	P3	P4	P5	P6	P7	P8	P9	P10	P11	P12
P1	*	**0.74**	**0.69**	**0.80**	**0.79**	0.33	0.40	0.32	0.40	**0.57**	**0.67**	**0.69**
P2		*	**0.59**	**0.71**	**0.83**	0.33	0.48	**0.50**	0.48	0.48	**0.58**	**0.60**
P3			*	**0.67**	**0.64**	0.47	**0.60**	**0.53**	**0.60**	**0.57**	**0.53**	**0.62**
P4				*	**0.81**	0.38	**0.50**	0.35	**0.50**	**0.63**	**0.65**	**0.73**
P5					*	0.42	0.45	0.48	0.45	**0.60**	**0.69**	**0.71**
P6						*	**0.55**	**0.60**	**0.55**	0.21	0.19	0.20
P7							*	**0.62**	**0.86**	0.36	0.25	0.35
P8								*	**0.62**	0.19	0.17	0.18
P9									*	0.27	0.25	0.35
P10										*	**0.81**	**0.84**
P11											*	**0.85**
P12												*

**Table 6:** Adult female mosquitoes screened for natural infection with YFV, all of which tested negative. Mosquitoes were collected with BG-Sentinel and protected human attraction at the Centro de Promatologia do Rio de Janeiro from December 2018 to December 2019.

Mosquito Species	Number of Tested Specimens
*Aedes albopictus*	4
*Aedes scapularis*	73
*Aedes terrens*	7
*Hg. janthinomys/capricornii*	57
*Hg. leucocelaenus*	116
*Culex* sp.	2
*Runchomyia* sp.	5
*Sabethes albiprivus*	1
*Sabethes chloropterus*	14
*Wyeomyia (Pho.)* sp.	8
Total	287
